# Aberrant pregnancy-associated plasma protein-A expression in breast cancers prognosticates clinical outcomes

**DOI:** 10.1038/s41598-020-70774-9

**Published:** 2020-08-13

**Authors:** Prashanth Prithviraj, Matthew Anaka, Erik W. Thompson, Revati Sharma, Marzena Walkiewicz, Candani S. A. Tutuka, Andreas Behren, George Kannourakis, Aparna Jayachandran

**Affiliations:** 1 Cancer Immunobiology Laboratory, Olivia Newton-John Cancer Research Institute, Heidelberg, VIC Australia; 2 Department of Medicine, University of Melbourne, Victoria, Australia; 3 Fiona Elsey Cancer Research Institute, Ballarat Technology Park- Central Suite 23, 106-110 Lydiard St Sth, Ballarat, VIC 3350 Australia; 4 Federation University Australia, Ballarat, VIC Australia; 5 Department of Medicine, University of Alberta, Alberta, Canada; 6 Institute of Health and Biomedical Innovation, Queensland University of Technology (QUT), Brisbane, Australia; 7 Translational Research Institute, Woolloongabba, Australia; 8 School of Cancer Medicine, La Trobe University, Victoria, Australia; 9 Gallipoli Medical Research Institute and The University of Queensland, Brisbane, Australia

**Keywords:** Breast cancer, Breast cancer

## Abstract

Elevated levels of pregnancy-associated plasma protein-A (PAPP-A) have been implicated in the pathogenesis of various malignancies, including breast cancers. Breast cancer is one of the most frequent carcinomas and is the second most common cancer type detected in women of child-bearing age. Throughout pregnancy PAPP-A is produced and secreted by the placental syncytiotrophoblast cells; co-incidentally pregnancy-associated breast cancers often have an aggressive clinical course. The components of the PAPP-A/IGF axis was assessed in a panel of breast cancer cell lines. Using neutralising antibodies the impact of PAPP-A/IGF axis on cell motility was evaluated. PAPP-A was expressed in four of the twelve breast cancer cell lines tested. Blocking PAPP-A and IGFBP4 with neutralising antibodies significantly decreased motiliy of MDA-MB-231 cells. Upregulation of PAPP-A expression in breast tumours resulted in a trend towards worse overall survival. Notably, PAPP-A expression also positively correlated with epithelial-to-mesenchymal transition markers. In conclusion, these results indicate that PAPP-A plays an important role in breast cancer progression and it may be a promising therapeutic target in breast cancer.

## Introduction

Breast cancer is a common malignancy, and prognosis of metastatic disease is significantly worse; thus, there is a pressing need for additional treatment strategies to impede progression of the disease. Breast cancer is the second most common cancer type detected in women of child-bearing age, and recurrence in pregnancy-associated breast cancer is correlated with worse outcome^1^. Triple-negative breast cancers (negative for expression of the oestrogen receptor [ER], the progesterone receptor [PR], and Her2) account for 10–20% of breast cancers, and have been reported to be more aggressive and associated with poorer survival than Her2 or hormone receptor-positive breast cancers^2^. Notably, although hormone receptor-positive tumours far outweigh the triple-negative subtype in general, and hormone levels change substantially during pregnancy, the triple-negative subtype of breast cancer is encountered more often than hormone receptor-positive tumours in pregnancy, suggesting an alternative mechanism of cancer promotion in pregnancy^3^.

The metalloproteinase, pregnancy-associated plasma protein-A (PAPP-A) cleaves the insulin-like growth factor binding protein 4 (IGFBP4) and insulin-like growth factor 1 (IGF-1) complex^4^. The proteolytic activity of PAPP-A leads to an increase in local free IGF-1 and activitation of IGF signalling pathway that has a pro-tumorigenic role in cancers^4^. PAPP-A was first identified in the plasma of pregnant women in 1974 and is secreted by placental syncytiotrophoblast cells, subsequently released into the maternal circulation. Secreted PAPP-A concentration rise exponentially in the first trimester. Its levels continue to rise throughout pregnancy until delivery, with plasma levels of PAPP-A increasing by a factor of 150 during pregnancy as compared with the non-pregnant state^4^. Notably, not only have high PAPP-A concentrations been detected in plasma obtained from pregnant women, but also in a number of pathological processes^4–8^.

Of relevance, PAPP-A plays an important role in regulating the availability of active IGF in cancers^9,10^. In stage I breast cancers, PAPP-A immunohistochemical staining positivity correlates with early recurrence^11^. PAPP-A has also been reported to be a predictive marker of early recurrence in both ER-positive and ER-negative tumours^11^. Components of the IGF pathway have been investigated as potential biomarkers in breast cancer. IGF-1 receptor (IGF-1R) expression correlates with poor survival and is thought to have a possible role as a prognostic marker, with higher concentrations of IGF-1 noted in the serum of breast cancer patients^12^. Nielsen et al. detected overexpression of IGF-1R in 87% of 930 patients with primary breast cancer^13^.

Given that increasing levels of PAPP-A provide a plausible biological mechanism for the link between pregnancy and progression and relapse of breast cancer, we hypothesised that PAPP-A can contribute to the progression of breast cancer. To this end, we evaluated the expression and function of PAPP-A and IGF axis components in breast cancer cell lines. We evaluated PAPP-A expression in clinical breast cancer samples, and examined its relationship with clinicopathological characteristics and survival outcome in patients with breast cancer. We also examined the role of PAPP-A in breast cancer cell proliferation and motility.

## Results

### PAPP-A is expressed in breast cancer patient tumours

Immunohistochemical staining was performed on breast cancer tissue microarrays (TMAs). The study cohort comprised 45 female patients with breast cancer. Table 1 depicts the clinical and pathological characteristics of the patients and the information available for analysis, including histopathological diagnosis, tumour grade (three grades that determine the histological phenotype as compared with normal), T stage (extent of primary tumour), and receptor (ER, PR, Her2) status. Lymph node status provides valuable information with regard to the ability of cancer cells to metastasise and is routinely considered prior to formulating a patient management plan. Information on lymph node status was available for 38 patients. Thirty-nine patients were analysed for lymphovascular invasion, which is an indicator of increased metastatic capability.

**Table 1:** Clinicopathological characteristics and PAPP-A immunoreactivity of 45 breast cancer patients.

Characteristic	Total (n)	n (%)
**Histopathology**	45	
Invasive ductal carcinoma		34 (77%)
Metaplastic carcinoma		2 (4%)
Adenoid cystic carcinoma		2 (4%)
Pleomorphic invasive lobular carcinoma		1 (2%)
Atypical medullary carcinoma		2 (4%)
Poorly differentiated carcinoma		4 (9%)
**Receptor status**	41	
**Oestrogen receptor**		
Positive		5 (13%)
Negative		36 (87%)
**Progesterone receptor**		
Positive		4 (9%)
Negative		37 (91%)
**Her2 status**		
Positive		1 (3%)
Negative		40 (97%)
**Grade**	42	
1		1 (2%)
2		5 (12%)
3		36 (86%)
**Tumour staging**	39	
T1 (< 2 cm)		17 (43%)
T2 (2–5 cm)		22 (52%)
T3 (> 5 cm)		2 (5%)
**Lymph node involvement**	38	
Yes		18 (47%)
No		20 (53%)
**Lymphovascular invasion**	39	
Positive		19 (49%)
Negative		20 (51%)
**PAPP-A immunohistochemistry**	45	
3+		7 (15%)
2+		12 (27%)
1+		7 (15%)
0		19 (43%)

**Figure 1:**
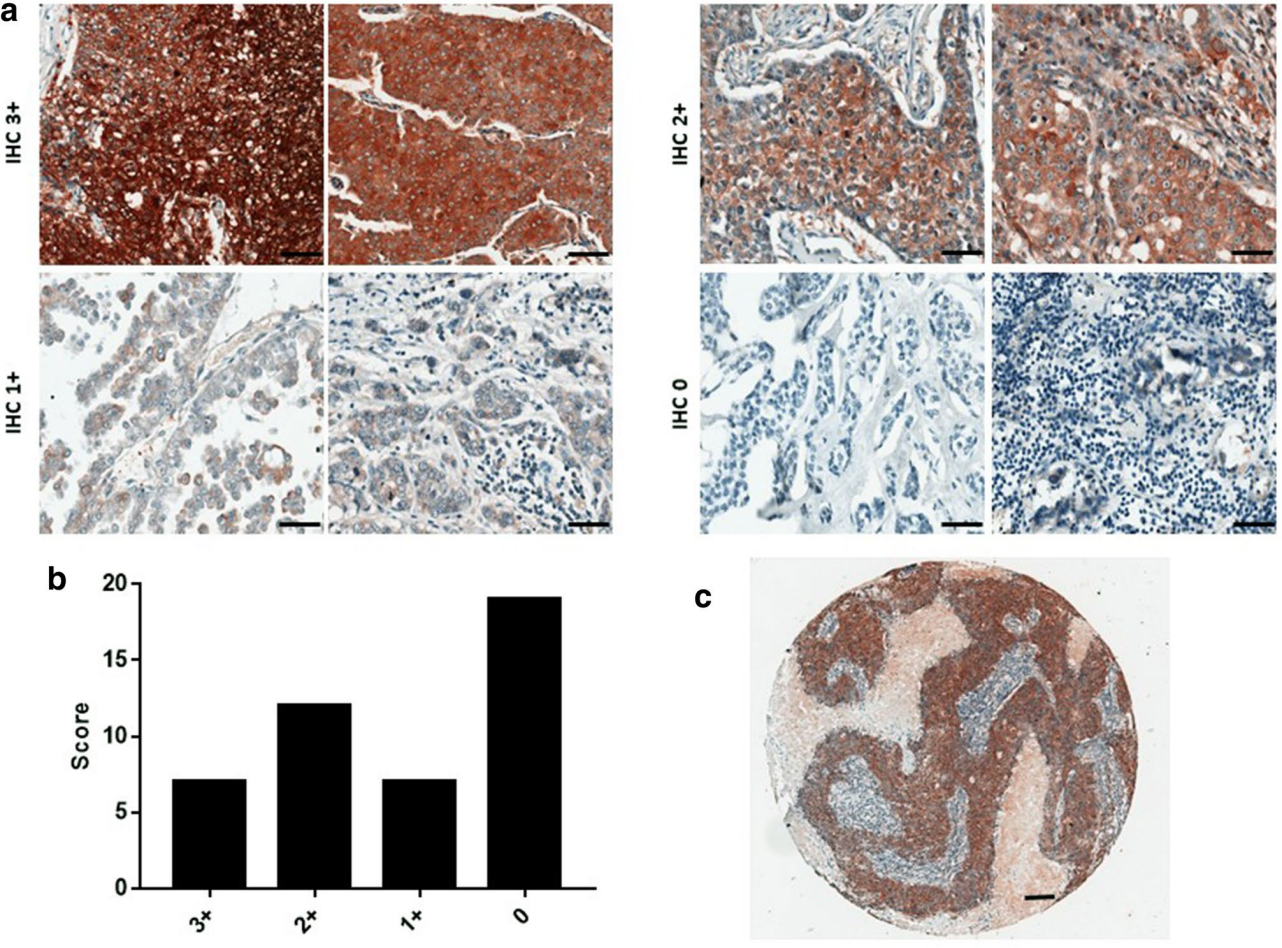
PAPP-A expression in breast cancer tissue microarray samples. (**a**) Representative images of PAPP-A expression in breast cancer TMAs. Positive PAPP-A immunostaining was graded as IHC 3+, 2+, or 1+. IHC 0 was graded for samples negative for PAPP-A immunostatining. Scale bar 50 µm. (**b**) Graph shows number of tumours scored. (**c**) Representative image of a core within the breast cancer TMA. Scale bar 150 µm.

TMAs consisted predominantly of triple-negative breast cancers, with more than 80% of tumours being negative for ER, PR, and Her2. The expression of PAPP-A was examined in these patient tumour samples. Patient cores were in triplicate and scored 3+, 2+, 1+, or 0 based on high, medium, low, and negative staining, respectively. As there were multiple patient cores in each TMA, scores from staining were averaged, and this score was used for further analysis. Expression of cytoplasmic and membranous PAPP-A was detected at variable intensity in breast cancer TMAs. Nineteen (43%) were negative and 26 (57%) were positive for PAPP-A expression. Seven tumours (15%) stained 3+, and twelve (27%) and seven (15%) stained 2+ and 1+, respectively (Fig. 1a–c).

### Expression of PAPP-A in breast cancer: relationship with clinicopathological characteristics

The Fisher's Exact and chi-square tests were used as statistical tools to investigate the relationship between expression of PAPP-A and pathological parameters (Table 2). Breast tumours were designated a positive (3+/2+) or negative (0/1+) score as the PAPP-A staining intensities of IHC 3+ and 2+ were very high compared with the very weak IHC 1+ staining. A two-tailed *P*-value of less than 0.05 was considered to be statistically significant. There was no significant relationship between PAPP-A expression and parameters such as grade, T stage, lymph node status or lymphovascular invasion, as outlined in Table 2. However, patients with high-grade tumours showed a strong trend towards increased PAPP-A expression (*P* = 0.06), and a similar trend was noted in lymph node status, where higher expression was noted in patients positive for lymph node involvement. Most PAPP-A staining positivity was noted in T2 stage tumours (size 2–5 cm).

**Table 2:** Association between PAPP-A staining and clinicopathological characteristics in breast cancer patients (PAPP-A-positive, 3+/2+; PAPP-A-negative, 0/1+.

Tumour characteristics	PAPP-A-positive n (%)	PAPP-A-negative n (%)	*P*-value
**Grade**			0.06
1	0/16 (0%)	1/20 (5%)	
2	2/16 (13%)	3/20 (15%)	
3	14/16 (87%)	16/20 (80%)	
**Tumour staging**			–
T1 (< 2 cm)	0/16 (0%)	0/19 (0%)	
T2 (2–5 cm)	15/16 (93%)	18/19 (94%)	
T3 (> 5 cm)	1/16 (7%)	1/19 (6%)	
**LN involvement**			0.67
Yes	8/15 (53%)	11/22 (50%)	
No	7/15 (47%)	11/22 (50%)	
**LVI**			0.2
Positive	7/15 (46%)	11/20 (55%)	
Negative	8/15 (54%)	9/20 (45%)	

LN, lymph node; LVI, lymphovascular invasion.

### Aberrant expression of PAPP-A associated with poor outcome in breast cancer patients

**Figure 2:**
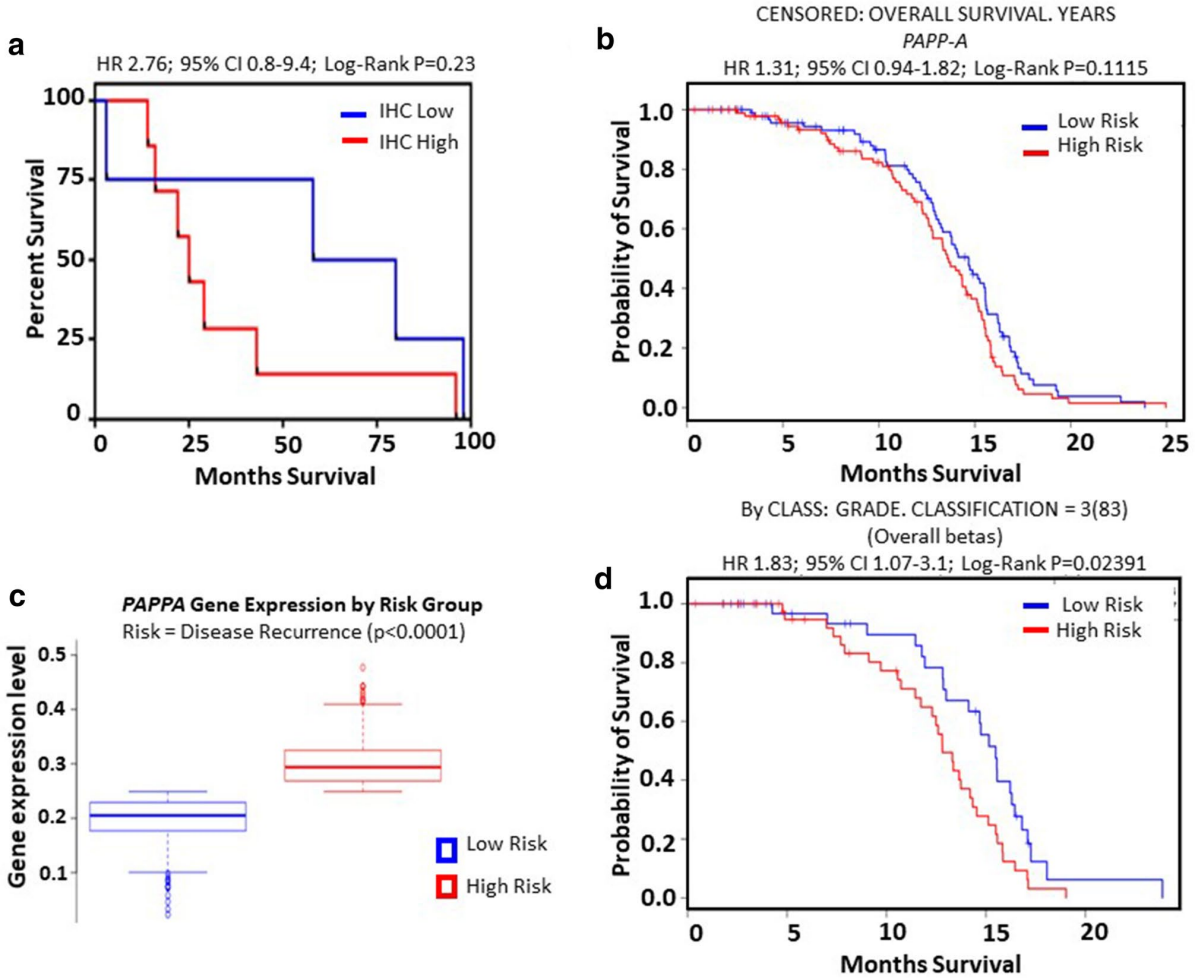
Effect of PAPP-A on overall survival and disease recurrence in breast cancer. (**a**) Kaplan–Meier estimates of overall survival (*P* = 0.23) as a function of expression of PAPP-A in breast cancer. (**b**) Overall survival (*P* = 0.11) as a function of expression of PAPP-A in breast cancer from a meta-analysis dataset. (**c**) *PAPP-A* expression showed a significant association with a high-risk prognostic score (*P* < 0.005), the risk being that of relapse in breast cancer patients from the meta-analysis dataset. (**d**) Overall survival (*P* = 0.02) as a function of expression of PAPP-A in breast cancer from dataset of 83 patients.

Of the 45 tumours stained by PAPP-A IHC, only a limited number came from patients with information on survival outcome. Thirteen tumours were analysed for effect of expression of PAPP-A on clinical outcome. As the PAPP-A staining intensities of IHC 3+ and 2+ were very high compared with the very weak IHC 1+ staining, tumours were classified as positive (3+/2+) or negative (0/1+). Expression of PAPP-A did not affect overall survival (*P* = 0.23). A Cox proportional regression hazard model was used to calculate the risk of death for a patient, with time to recurrence and death as endpoints and PAPP-A being the predictive variable. Hazard ratios are as outlined in Fig. 2a. Although a limited number of patients were examined, a strong trend of worse survival was noted in the PAPP-A-positive arm, with a median survival of 25 months as compared with 69 months in the PAPP-A-negative arm, with a hazard ratio of 2.76 and a wide confidence interval. Analysis of a larger cohort is necessary to determine the effect of PAPP-A on survival.

We then extended our initial findings to a larger cohort from a meta-analysis that included molecular profiling for nine datasets (n = 1574) (https://bioinformatica.mty.itesm.mx:8080/Biomatec/SurvivaX.jsp). Overall survival in this cohort again showed a strong trend associating PAPP-A expression with worse survival (*P* = 0.11, hazard ratio 1.31; confidence interval 0.94–1.82) (Fig. 2b). On further analysis, *PAPP-A* gene expression forms a component of the high-risk group (*P* < 0.005), with the risk being disease recurrence (Fig. 2c). High PAPP-A expression associated significantly (*P* = 0.02, hazard ratio 1.83; confidence interval 1.07–3.1) with poorer overall survival in grade 3 tumours compared with grade 1 or 2 tumours (Fig. 2d).

**Figure 3:**
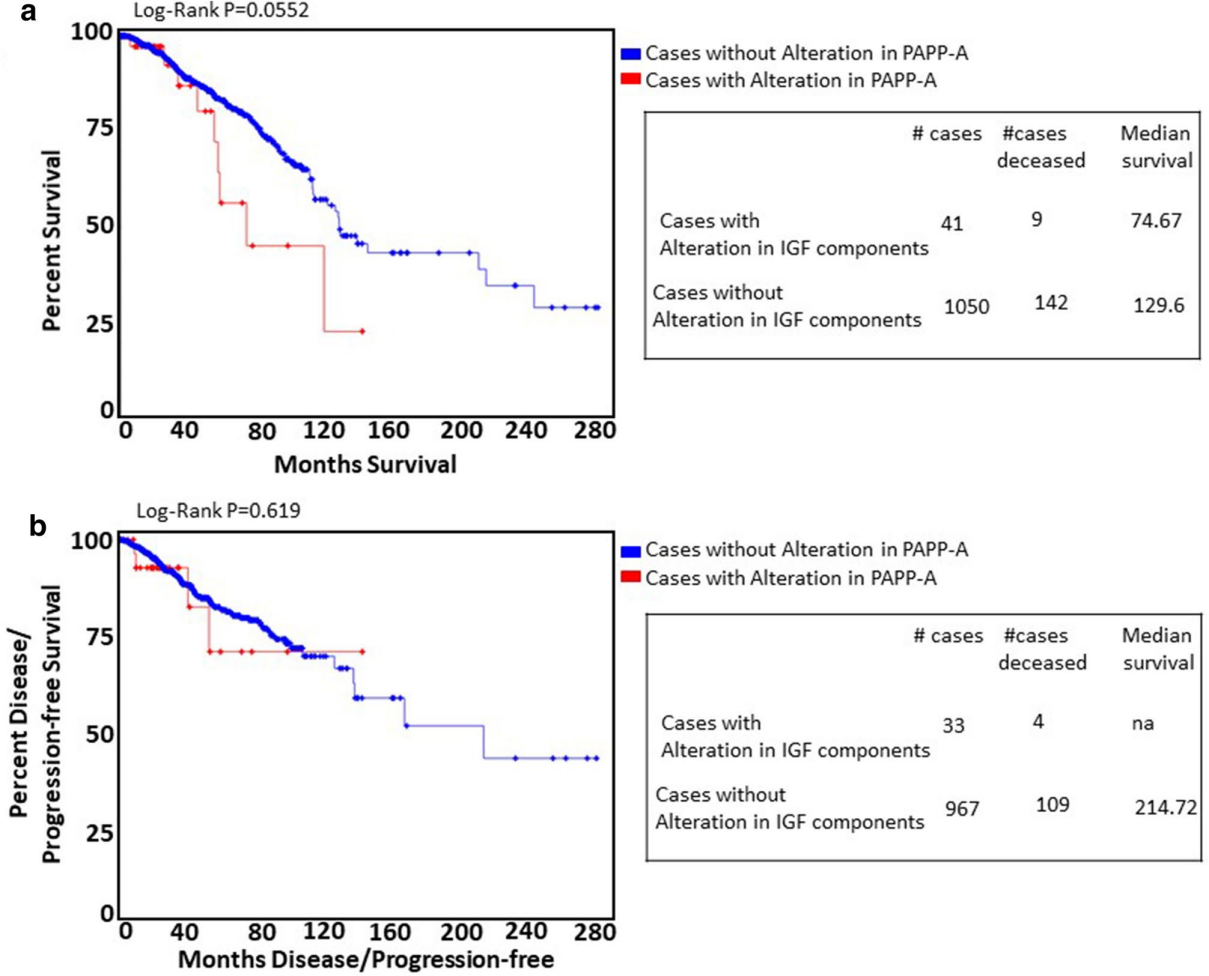
PAPP-A in breast cancer: analysis of TCGA dataset. (**a**) Kaplan–Meier estimates of overall survival (*P* = 0.05) as a function of expression of PAPP-A in breast cancer (n = 1,091). (**b**) Kaplan–Meier estimates of progression-free survival (*P* = 0.619) as a function of expression of PAPP-A in breast cancer (n = 1,000).

We next analysed the publically available breast cancer genomics datasets at cBioportal for alterations in PAPP-A (Supplementary Table 1). Coding mutations were rare, occurring in 0–1.8% of samples. Alterations in gene expression were most common, occurring in 2.9–5% of samples, with up-regulation the most common change. The Cancer Genome Atlas (TCGA) datasets provided the only available RNA-seq data with associated survival information. In the provisional TCGA dataset, up-regulation of *PAPP-A* expression resulted in poorer overall survival in this dataset containing 1,090 patients (*P* = 0.0552). Median survival of patients with alteration in *PAPP-A* expression was 74.67 months compared with 129.6 months in patients without alteration in *PAPP-A* (Fig. 3a). There was no significant difference in progression free survival (*P* = 0.619) (Fig. 3b). Taken together, PAPP-A seems to confer worse survival in breast cancers in general, an effect which may be amplified by the high levels of circulating PAPP-A during pregnancy, resulting in the observed increased risk of disease recurrence in that context.

### Breast cancer cell lines express PAPP-A and components of the IGF axis

**Figure 4:**
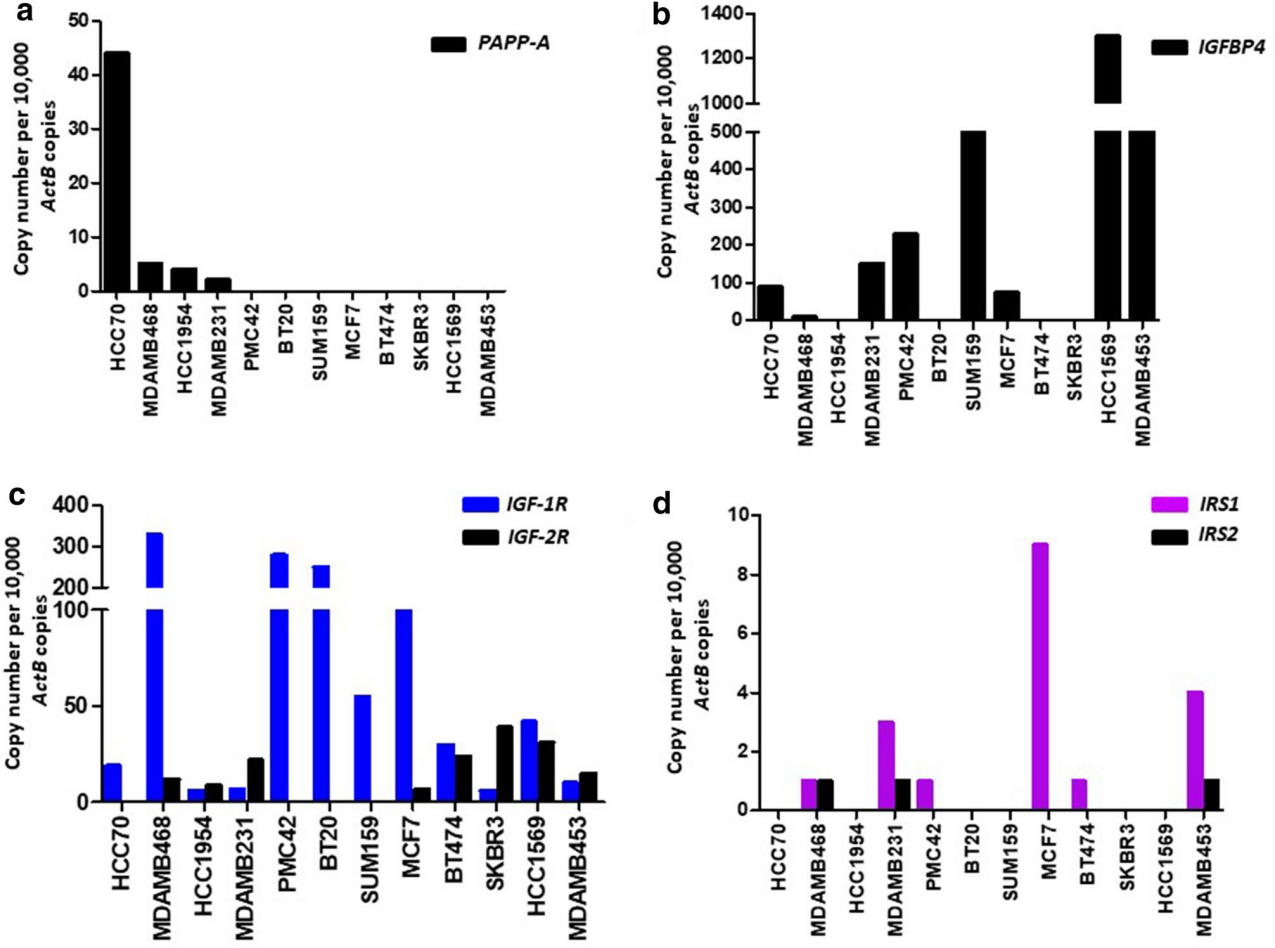
Expression of *PAPP-A* and components of the *IGF* axis in breast cancer cells. qRT-PCR analysis of expression of (**a**) *PAPP-A*, (**b**) *IGFBP4*, (**c**) IGF receptors (*IGF-1R* and *IGF-2R*), and (**d**) insulin receptor substrates (*IRS1* and *IRS2*) in breast cancer cell lines.

We examined *PAPP-A* mRNA expression by qRT-PCR in a panel of twelve human breast cancer cell lines. *PAPP-A* expression was noted in four cell lines HCC70, MDA-MB-468, HCC1954 and MDA-MB-231 (Fig. 4a). It has been reported that PAPP-A exerts its biological effect through cleavage of its primary substrate IGFBP4^14^. This in turn makes IGF-1 bioavailable at its receptor to enable local IGF action. We subsequently examined the expression of *IGFBP4* in the breast cancer cell lines and found it to be expressed in eight of the twelve cell lines examined. Three of the cell lines that expressed *PAPP-A* (HCC70, MDA-MB-468 and MDA-MB-231) also showed *IGFBP4* expression, while HCC1954 only expressed *PAPP-A* and not *IGFBP4* (Fig. 4b). Next, we assessed the expression of other important components of the IGF axis including IGF receptors (*IGF-1R* and *IGF-2R*) and Insulin receptor substrates (*IRS-1* and *IRS-2*). *IGF-1R* was expressed in all cell lines, while *IGF-2R* expression was detected in eight cell lines (Fig. 4c). *IRS-1* was expressed in six cell lines and *IRS-2* was expressed in MDA-MB-468, MDA-MB-231 and MDA-MB-453 (Fig. 4d).

### Effect of blocking PAPP-A/IGF axis components on breast cancer cell proliferation, migration and invasion

**Figure 5:**
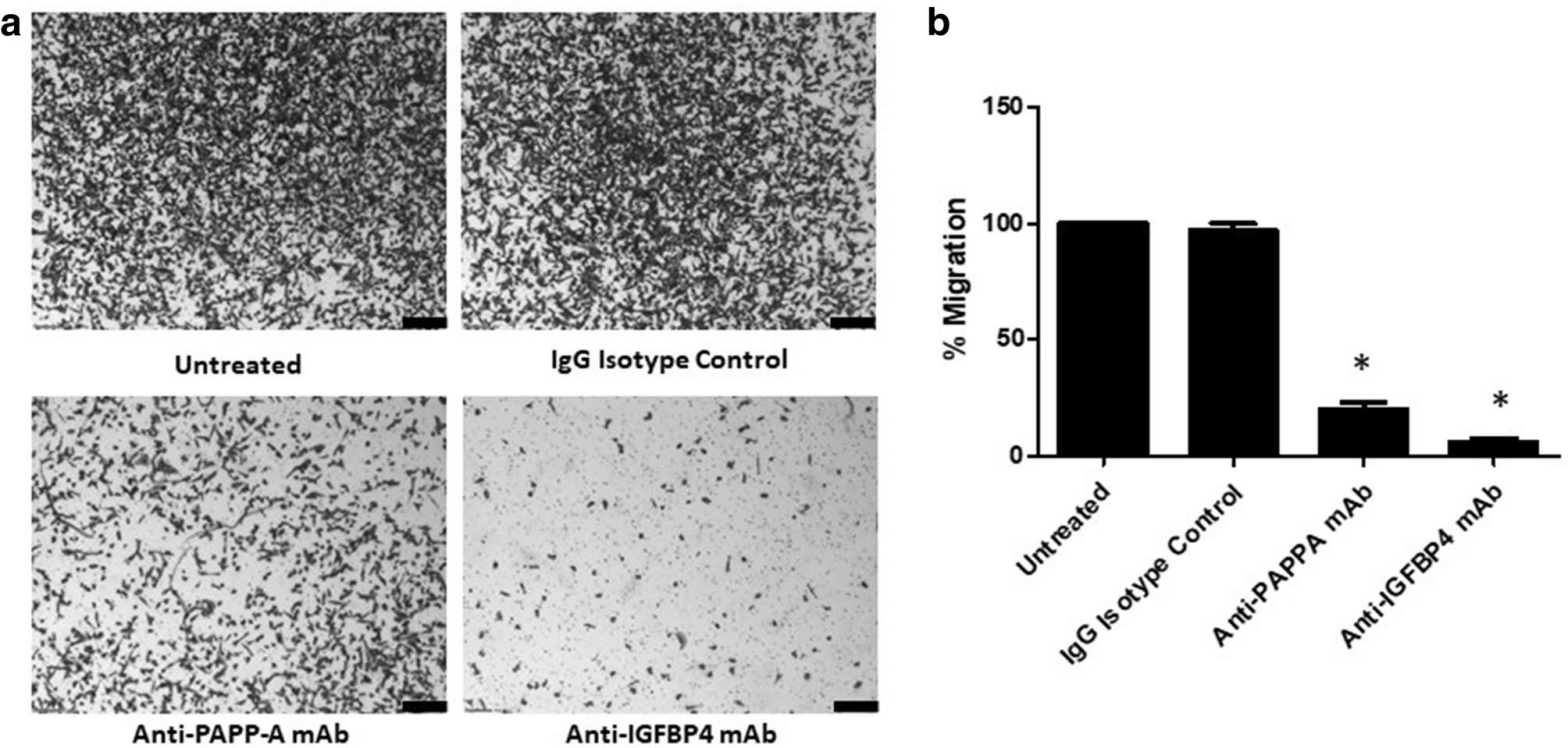
Inhibition of migration of a breast cancer cell line with neutralising antibodies to PAPP-A and IGFBP4. (**a**) Migratory ability of MDA-MB-231 breast cancer cells (using the Boyden chamber assay) after treatment with control anti-IgG, anti-IGFBP4 antibody or anti-PAPP-A antibody was tested and the images captured. Scale bar 200 μm. (**b**) The graphs show the percentage of migratory cells counted. Values are mean +/− SEM of three experiments in triplicate (*P < 0.05).

**Figure 6:**
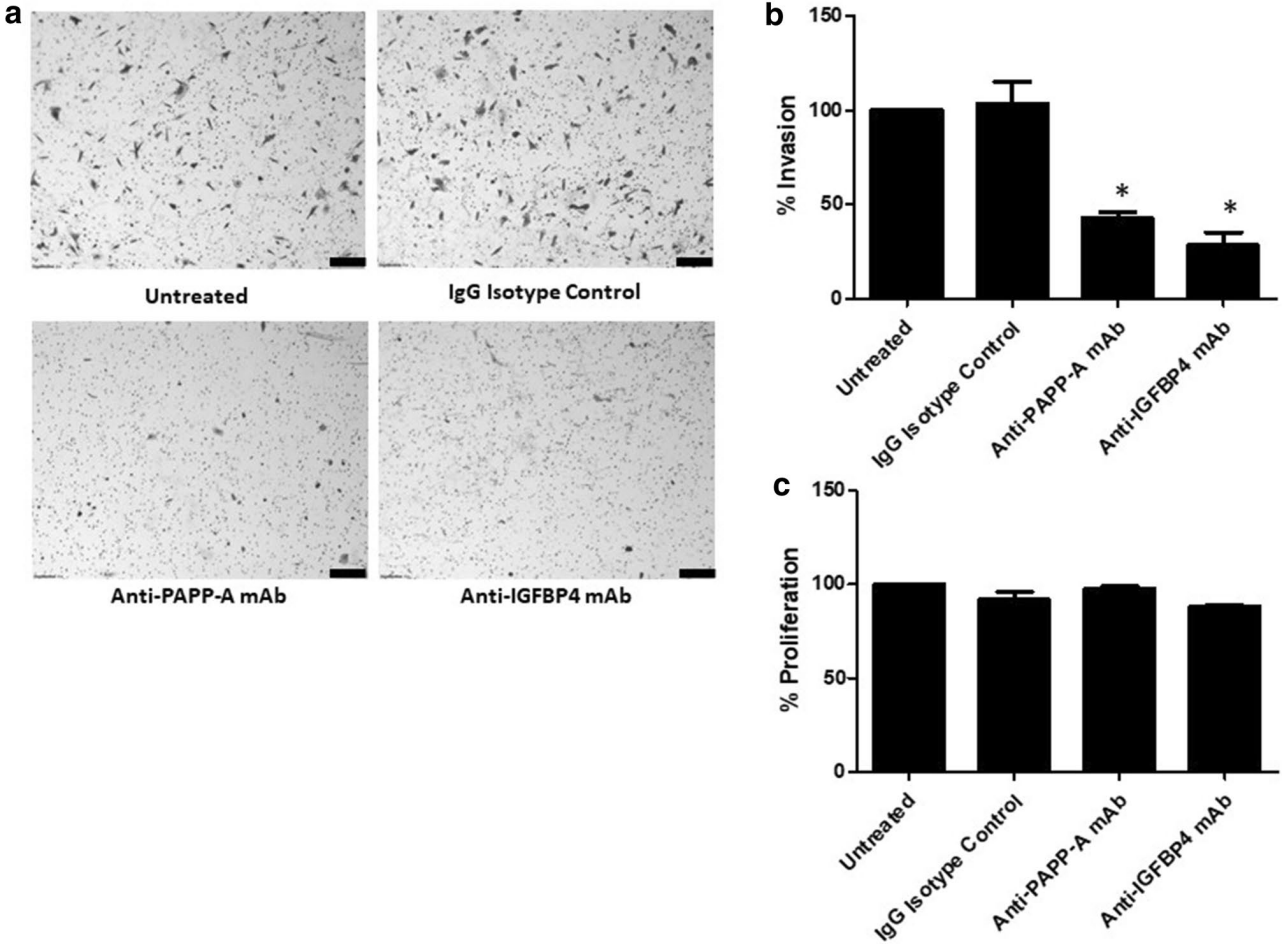
Effect of neutralising antibodies to PAPP-A and IGFBP4 on invasion and proliferation of breast cancer cells. (**a**) Invasive ability of MDA-MB-231 breast cancer cells after treatment with control anti-IgG, anti-PAPP-A antibody or anti-IGFBP4 antibody was tested and the images captured. Scale bar 200 μm. (**b**) The graphs show the percentage of invasive cells counted. (**c**) The graphs show the percentage of proliferation of MDA-MB-231 breast cancer cells after treatment with control anti-IgG, anti-PAPP-A antibody or anti-IGFBP4 antibody. Values are mean +/− SEM of three experiments in triplicate (*P < 0.05).

We have previously reported the pro-migratory role of PAPP-A in melanoma^8^. We next sought to investigate whether PAPP-A shared this pro-migratory role in breast cancer or if this role is unique to melanoma. When the breast cancer cell line MDA-MB-231 was co-incubated with an anti-PAPP-A antibody, there was a significant decrease in the migratory ability (Fig. 5a,b). To further examine whether other components of the IGF axis had an effect on the migratory ability of breast cancer cells, neutralising antibodies to IGFBP4 were co-incubated with MDA-MB-231. Anti-IGFPB4 antibody significantly reduced the migratory capability of these breast cancer cells (Fig. 5a,b). Antibody treatment of the breast cancer cell line MDA-MB-231 with anti-PAPP-A and anti-IGFBP4 antibodies decreased the invasive ability of these cells (Fig. 6a,b). The effect of the neutralising antibodies to PAPP-A and IGFBP4 on cell proliferation was evaluated on MDA-MB-231. These antibody treatments exerted no effect on the viability of MDA-MB-231 cells (Fig. 6c). The diminished motility of MDA-MB-231 was similar to the abrogation of motility previously noted with the anti-PAPP-A and anti-IGFBP4 monoclonal antibodies in melanoma cells^8^. This was a proof-of-concept experiment to confirm that the PAPP-A/IGF axis is important in breast cancer and that motile ability can be attenuated by modulation of components of the IGF axis. This demonstrates that indirect inhibition of IGF signalling has a potentially important role in non-melanomatous cancers.

### Up-regulation of IGF axis components is associated with worse progression free survival

**Figure 7:**
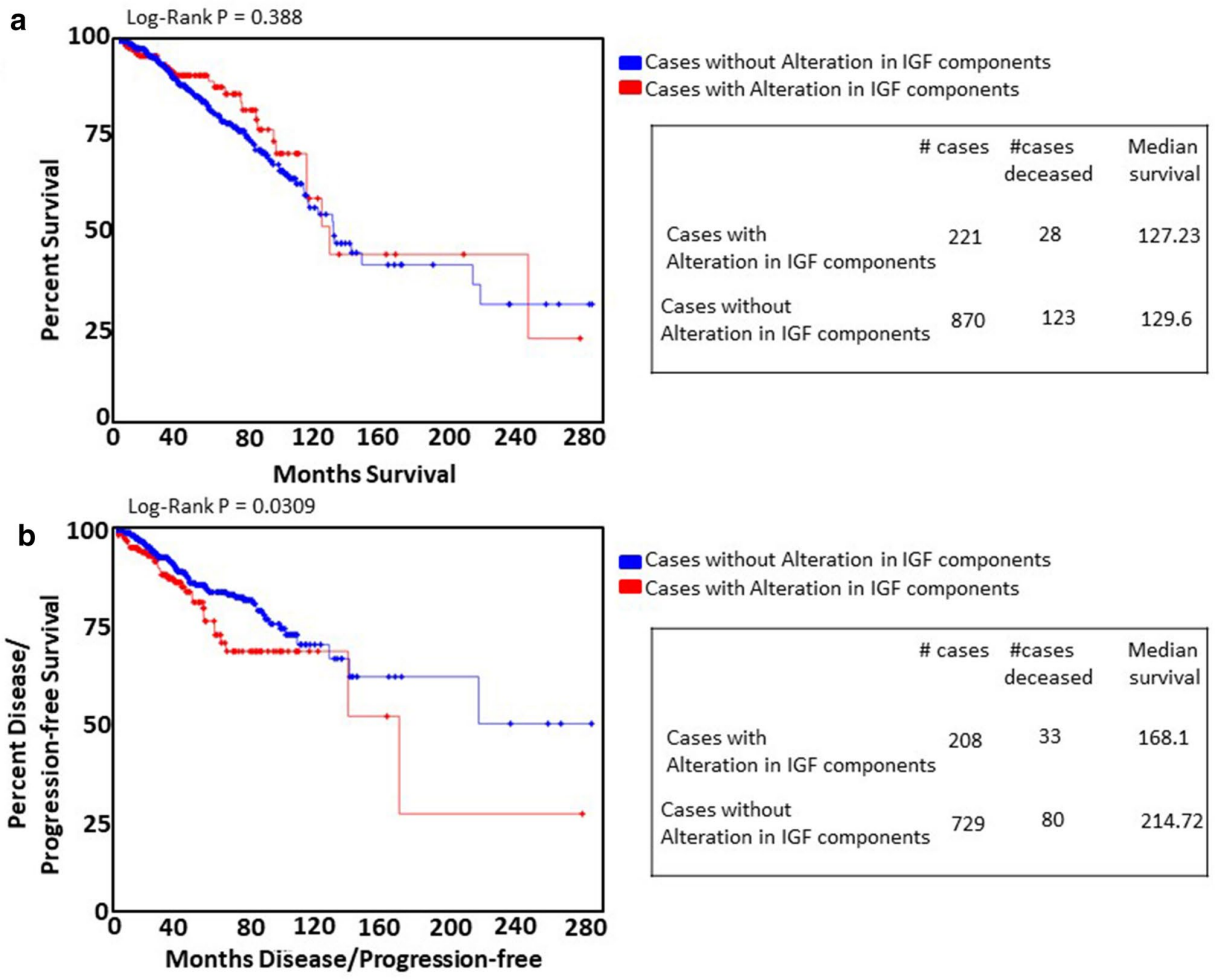
IGF components in breast cancer: analysis of TCGA dataset. (**a**) Kaplan–Meier estimates of overall survival (*P* = 0.3) as a function of expression of IGF components in breast cancer (n = 1,091). (**b**) Kaplan–Meier estimates of progression-free survival (*P* = 0.0309) as a function of expression of IGF components in breast cancer (n = 937).

We interrogated the TCGA provisional dataset for association of gene expression alterations in the IGF signalling components analysed above (IGF-1R, IFG-2R, IGFBP4, IRS-1, IRS-2) with survival in breast cancer (Fig. 7). 223 of 1,093 (20%) had altered expression of one or more molecules, the majority of which (197) were up-regulation. While there was no significant difference in overall survival, progression-free survival was significantly reduced in patients with upregulated expression of the gene set components (168.1 vs. 214.72 months; n = 1,000, *P* = 0.0309) (Fig. 7a,b).

### PAPP-A expression positively correlates with epithelial-to-mesenchymal transition markers in breast cancers

**Figure 8:**
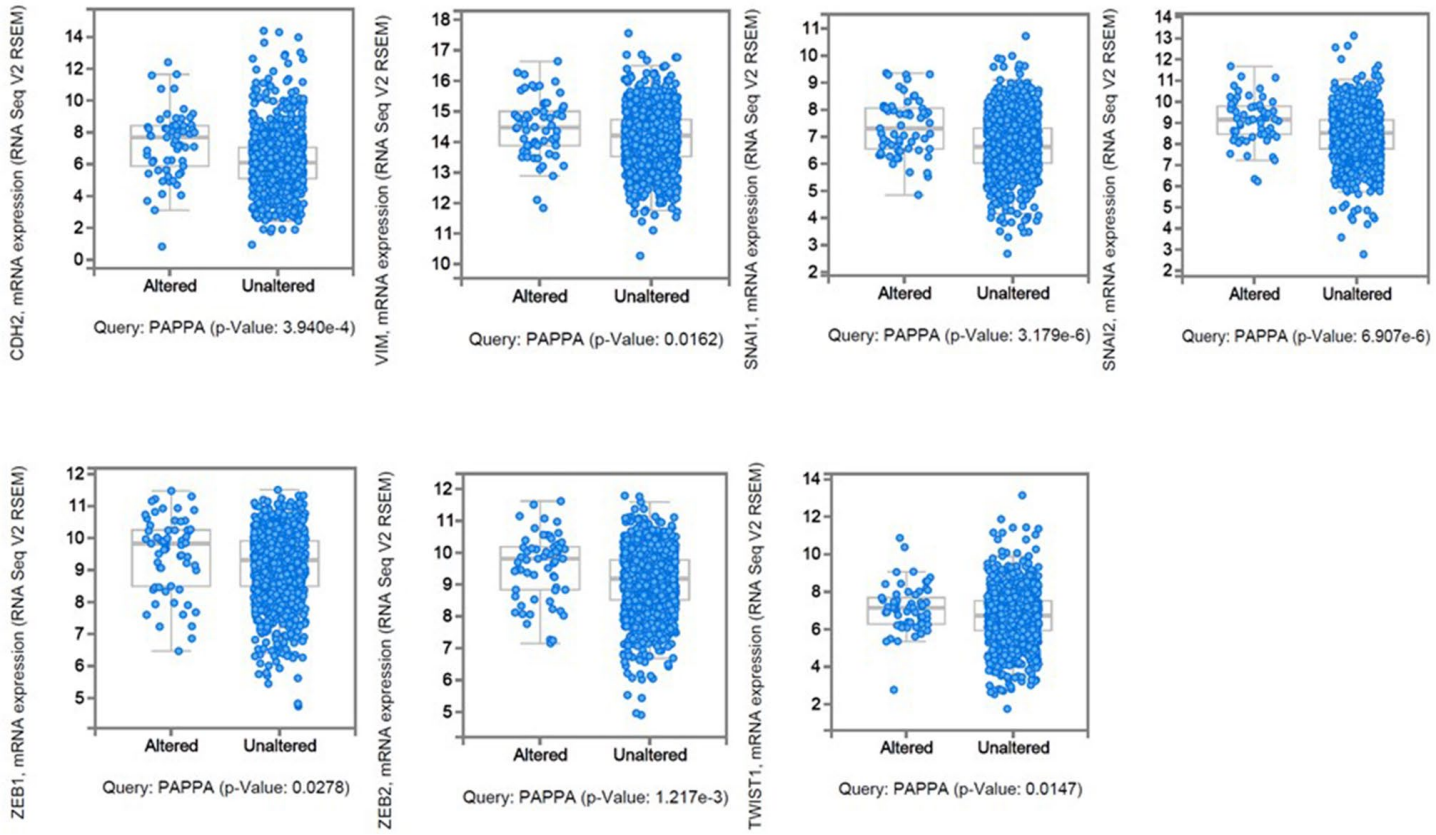
PAPP-A expression is associated with a mesenchymal phenotype in breast cancers. Enrichment of mesenchymal markers CDH2, VIM, SNAI1, SNAI2, ZEB1, ZEB2 and TWIST1 with PAPP-A is shown in a breast cancer dataset from TCGA.

Epithelial-to-mesenchymal transition (EMT) describes a reprogramming of epithelial cells that results in them acquiring a mesenchymal phenotype with increased motile abilities^15^. We investigated if the PAPP-A/IGF axis correlates with EMT phenotype, similar to our previous observation in melanoma^8^. Mutual exclusivity data from the n = 1,105 breast cancer TCGA dataset revealed that PAPP-A has a significant tendency towards co-occurrence with mesenchymal markers Vimentin (VIM), SNAI1 (Snail), SNAI2 (Slug), Zeb1, Zeb2 and Twist1 (Table 3). Furthermore, analysis of RNAseq data validated that *PAPP-A* was significantly co-expressed with mesenchymal markers (Fig. 8). The data indicates that *PAPP-A* expression is associated with the aggressive mesenchymal phenotype in breast cancer patients.

**Table 3:** PAPP-A shows tendency towards co-occurrence with EMT markers.

GENE A	GENE B	*P*-value	Log odds ratio	Association
*PAPP-A*	*VIM*	< 0.001	1.390	Tendency towards co-occurrence
*PAPP-A*	*ZEB1*	0.001	1.319	Tendency towards co-occurrence
*PAPP-A*	*ZEB2*	0.001	1.500	Tendency towards co-occurrence
*PAPP-A*	*SNAI1*	0.011	0.830	Tendency towards co-occurrence
*PAPP-A*	*TWIST2*	0.027	1.088	Tendency towards co-occurrence
*PAPP-A*	*TWIST1*	0.039	1.105	Tendency towards co-occurrence

**Figure 9:**
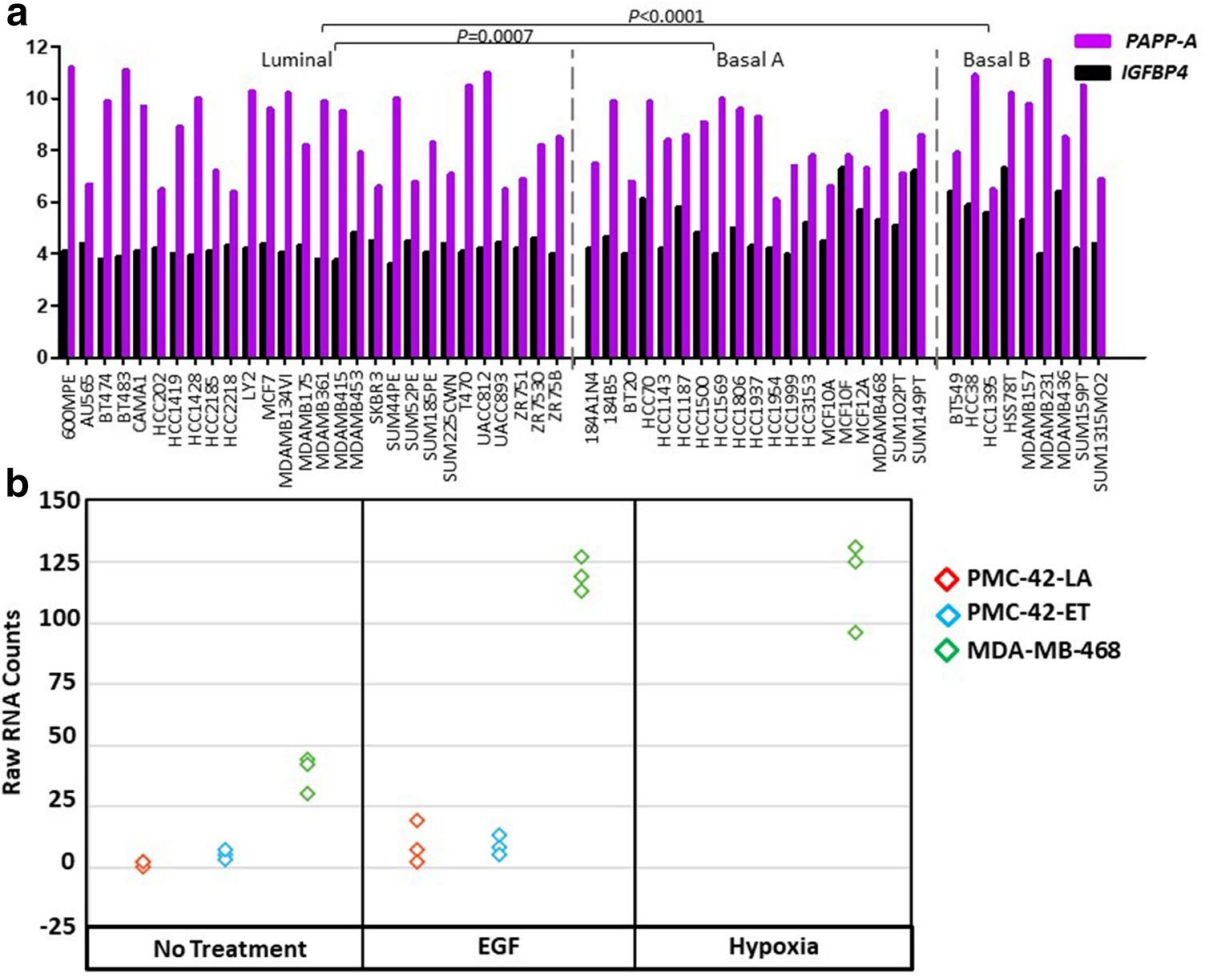
*PAPP-A* and *IGBP4* expression levels in 51 breast cancer cell lines. (**a**) Median centred mRNA expression levels for *PAPP-A* and *IGFBP4* are shown on a log scale. For genes represented by multiple probesets on the arrays, the probeset with the greatest standard deviation across samples was selected. *PAPP-A* expression (Tukey's multiple comparisons test) in Basal A versus Luminal *P* = 0.0007, Basal B versus Luminal *P* < 0.0001, while Basal A versus Basal B is non-significant (*P* = 0.2495). *IGFBP4* expression levels were similar across all three cell subgroups (One-way ANOVA *P* = 0.3029). (**b**) PAPP-A levels increase upon exposure to EMT inducing agents in breast cancer cell lines. Raw RNA Counts of PMC42-ET, PMC42-LA and MDA-MB-468 cells untreated or treated with EGF or Hypoxia.

We next analysed a dataset (E-MTAB-181) of 51 human breast cancer cell lines profiled with Affymetrix HG-U133A chips for gene expression level of *PAPP-A* and EMT markers^16–18^. These breast cancer cell lines were organised into the three molecularly distinct subgroups namely Luminal, Basal A and Basal B^19^. Cell lines clustering to Basal B expressed mesenchymal gene products such as *VIM*, *N-Cadherin*, *fibronectin*, *Slug*, *Twist* and *Zeb1* and lacked epithelial marker expression (e.g. *E-Cadherin*). The Luminal cluster represents cell lines with epithelial attributes, while Basal B show the most mesenchymal expression patterns, and Basal A cell lines exhibit features of both Basal B and Luminal cell lines^16,20^. While PAPP-A was expressed across all the subgroups of breast cancer cell lines, the Basal B subgroup showed relative enrichment in *PAPP-A* expression, which was significant compared to the Luminal subgroup but not the Basal A subgroup. However, the Basal A subgroup was also significantly higher that the Luminal subgroup (One-way ANOVA for PAPP-A, *P* < 0.0001), and for Tukey's multiple comparisons test, Basal A versus Luminal *P* = 0.0007, Basal B versus Luminal *P* < 0.0001, while Basal A versus Basal B is non-significant (*P* = 0.2495) (Fig. 9a). *IGFBP4* expression was seen to a similar level across all three cell subgroups (One-way ANOVA *P* = 0.3029) (Fig. 9a). The data indicates that PAPP-A expression is associated with the motile mesenchymal phenotype in breast cancer cell lines.

Several systems have emerged in which EMT can be induced in human breast cancer cell lines. Stimulation of PMC42-LA and the parental PMC42 cells (PMC42-ET) cells with epidermal growth factor (EGF) leads to the induction of EMT with changes in EMT marker expression^16^. Induction of EMT with EGF stimulation in PMC42-LA and PMC42-ET was associated with slight upregulation of *PAPP-A* expression, however a marked elevation in PAPP-A expression was seen in MDA-MB-468 exposed to EGF (1.96-fold increase; *P*_(*FDR*)_ = 0.000555) or hypoxia (1.695-fold increase; *P*_(*FDR*)_ = 0.002260) (Fig. 9b).

## Discussion

Herein, we report the upregulation of PAPP-A expression in breast cancer patient tumours and cell lines. We observed that patients with high-grade tumours and positive lymph node status showed increased expression of PAPP-A. In breast cancer patients PAPP-A seems to confer worse survival with elevated risk of disease recurrence. Moreover, we could demonstrate that PAPP-A/IGF axis is important in breast cancer and that motile ability of breast cancer cells can be attenuated by modulation of components of the IGF axis. We observed an association of PAPP-A expression with mesenchymal phenotype in breast cancer cell lines and samples from breast cancer patients. Notably, in breast cancer cell lines we found upregulation of PAPP-A expression during EGF and hypoxia-induced EMT.

The deregulation of PAPP-A expression and secretion has been linked with tumour progression in various cancers including lung cancer, melanoma, ovarian cancer and mesothelioma^8,9,21,22^. Our finding that the expression of PAPP-A is a predictor of poor survival and early recurrence in breast cancer patients is consistent with previous reports demonstrating PAPP-A positive immunostaining as a predictor of early recurrence in patients with stage II breast cancer or stage I estrogen receptor negative breast cancer^23,24^. In contrast, PAPP-A is epigenetically silenced in invasive breast carcinoma and ductal carcinoma in situ (DCIS) by the strong hypermethylation of PAPP-A promoter^25^. Another study has linked the delivery of large infants to high risk of breast cancer due to the elevated maternal concentrations of E3 and PAPP-A and reduced concentrations of alpha-feto protein (AFP) during pregnancy^26^. It is conceivable that breast cancer may arise in the vast majority of cases with high PAPP-A levels during pregnancy. However, our study lacks information on the time frame of breast cancer development after pregnancy. Larger studies with patient pregnancy and breast cancer diagnosis timelines might clarify the impact of PAPP-A expression in pregnancy-associated breast carcinoma.

Herein, PAPP-A expression was not associated with clinical parameters including tumour grade, tumour stage, lymph node status, and lymphovascular invasion. Similarly, another report showed that PAPP-A was not associated with HER2, ER status or tumour grade^27^. However, this is not a reflection of PAPP-A’s effect on the rate of tumour growth. Recent functional studies suggest that PAPP-A facilitates a microenvironment which may be advantageous for tumour growth by locally altering the host’s immune and coagulation systems^24^. Furthermore, PAPP-A expression was higher in more aggressive Luminal B breast cancers than Luminal A, consistent with their increased proliferative index and worse survival^27^. Another study reported elevated expression of *PAPP-A* transcripts in breast cancer cell lines harbouring p53 mutation^28^. We reported that PAPP-A or IGFBP4 antibody-mediated neutralisation abrogated migration and invasion but not proliferation in breast cancer cells. Further studies examining the mechanistic and functional role of PAPP-A in breast cancers are warranted.

PAPP-A is a putative regulator of IGF1 and contributes to the local bioavailability of IGF1 via the cleavage of IGFBPs^10,29^. IGFBP4 is a primary target of PAPP-A. PAPP-A cleaves IGFBP4 only when it is associated with either IGF-1 or IGF-2 in an IGF-dependent manner. As a result, in the presence of PAPP-A, IGFBP4 behaves as an IGF donor^4,30,31^. Our observation that the expression of PAPP-A was linked with enhanced motility aligns with our previous study in melanoma demonstrating that PAPP-A had pro-migratory function^8^. Notably, blocking IGFBP-4 was also associated with decreased cell motility in breast cancer. We did not exhaustively study if the pro-migratory and pro-invasive role of PAPP-A and IGFBP4 is mediated by a proteolytic mechanism. The migratory and invasive function of PAPP-A in breast cancer may also be facilitated by other non-proteolytic mechanisms associated with its multi-domain structure. Previously, we have shown that PAPP-A was enriched in melanoma mesenchymal-like cells and noted a significant correlation of PAPP-A with EMT markers^8^. EMT has a pro-migratory role in breast cancer and there is increasing evidence of EMT in clinical breast cancer samples^16^. The breast cancer cell lines database utilised herein mirror molecular subtypes and pathways found in breast cancer tumours^17^. The database showed expression of PAPP-A in all the three subgroups. However, its expression was enriched in Basal B and few Basal A cell lines. Our qRT-PCR data revealed that *PAPP-A* showed heterogeneous expression across the 3 subgroups. *PAPP-A* was expressed in some Basal B (MDA-MB-231) and Basal A (HCC70, MDA-MB-468 and HCC1954) subgroups, while Luminal (MCF7, BT474, SKBR3, MDA-MB-453) subgroup did not express *PAPP-A*.

Previous studies have implicated the expression of IGF receptor to aggressiveness in breast carcinoma^32^. However IGF receptors as targets in clinical trials has not been promising due to the lack of predictive biomarkers for patient selection, IGF receptors being very similar to insulin receptors and severe hypoglycaemia as a consequence of altered activity of insulin^33^. Based on our observations, it is conceivable that evaluation of PAPP-A expression may represent a promising strategy for stratifying breast cancer patients who may clinically benefit from PAPP-A/IGF axis targeted drug therapies. Furthermore, the advantages of targeting PAPP-A in breast cancer is that it is more selective in blocking IGF receptor signalling with high specificity and low off-target effects due to PAPP-A’s more restricted expression profile in normal tissue^34^. The fact that PAPP-A functionally drives motility of melanoma and breast cancer in addition to regulating IGF activity during pregnancy strongly supports the involvement of common or overlapping networks in cancer and pregnancy. Thus follow-up studies may identify druggable targets suitable for therapeutic intervention. In conclusion, we present strong evidence that PAPP-A plays an important role in breast cancer progression.

## Methods

### Tissue culture

Eleven breast cancer cell lines representing varying stages of disease and sourced from A/Prof Elgene Lim, LaTrobe University, Melbourne. EWT sourced MDA-MB-468 from the ATCC via the Lombardi Cancer Centre, Georgetown University. Mycoplasma testing of cell lines was performed with MycoAlert test kit (Lonza Rockland, Inc., USA). Cells were seeded in RPMI1640 containing10% fetal calf serum. The relevant guidelines and regulations were followed while performing cell culture methods.

### Breast cancer patient samples

Written informed consent was collected from all donors for tissue collection and research as per the Austin Health Human Research Ethics Committee (AHHREC), Australia, approved protocol (H2012/04446). The relevant guidelines and regulations were followed while handling patient samples.

### qRT-PCR

The RNEasy kit (Qiagen, Germany) was utilised to obtain breast cancer cell line RNA. The High Capacity cDNA RT kit (Life Technologies, USA) was utilised for cDNA synthesis as previously described^8,35^. SYBR Green (Qiagen, Germany) was utilized for quantitative real-time RT-PCR (qRT-PCR). The primers sequences were previously reported^8^. These experiments were performed in accordance with the relevant guidelines and regulations.

### Immunohistochemistry and pathological evaluation

Immunohistochemistry was performed as previously described^8^. Briefly, following deparaffinisation and rehydration of paraffin embedded tissue slides, endogenous peroxidise activity was blocked with 3% Hydrogen peroxide. 10 mmol/L citrate buffer was used for antigen retrieval. Blocking reagent (Dako) was used to block nonspecific binding. 1.5 µg/mL concentration of PAPP-A antibody (Sigma Aldrich; HPA 001667) was used with overnight incubation at 4 °C. Secondary anti-rabbit antibody HRP (Dako) incubation was performed for 60 min^8^. 3-amino-9-ethylcarbazole (AEC) was used as the chromogen. Human placenta provided positive control staining for PAPP-A*.* Primary antibody substituted with the same concentration of rabbit IgG was used as a negative control. ScanScope XT (Aperio) was used to scan slides. Two independent investigators conducted immunohistochemical reactivity evaluations. PAPP-A expression was grouped into four grades: IHC 3+ , strong staining; IHC 2+, moderate staining; IHC 1+, weak staining and IHC 0, no staining. This method was performed in accordance with the relevant guidelines and regulations.

### Migration and invasion assays

Boyden chamber inserts were used to perform migration and invasion assays as previously described^8^. For invasion assays the Boyden chamber inserts were coated with Matrigel (Becton, Dickinson and Company, USA). 0.1% crystal violet solution (Sigma, USA) was used to stain the insert membrane, followed by analysis with Cell Sensi Software. A monochromatic Olympus camera was used to photograph cells. Migrated or invasive cells on the base of the insert were observed with 10X objective lens and counted from three random fields of view. Average of migrated or invaded cells was calculated from independent experiments repeated three times as previously described^36^. These methods were conducted in accordance with the relevant guidelines and regulations.

### Proliferation assay

In 96 well plates, 10,000 cells per well were seeded in triplicate. Cells were treated as indicated. The Water-soluble tetrazolium (WST-1) assay (Roche Applied Science, Germany) was conducted to measure relative cell numbers with a Bio-Rad iMark plate reader. This method was performed in accordance with the relevant guidelines and regulations.

### Analysis of publically available breast cancer genomics datasets

Analysis of publically available breast cancer genomics datasets was performed using cBioportal and SurvExpress^37,38^. Data shown are from analysis performed on March 28th 2018.

### Statistical analysis

All statistical comparisons of data sets were performed using Student’s two-tailed t-test, Anova or Kaplan–Meir survival analysis in Prism software version 5.00 (GraphPad Software Inc). The Fisher’s Exact and chi-square tests were used as statistical tools to investigate the relationship between expression of PAPP-A and pathological parameters. Statistical significance was set at *P* < 0.05. Cox's regression analysis was used to determine hazard ratios for overall survival. Kaplan–Meier analysis was performed to plot progression-free survival and overall survival along with curves to determine the relationship between PAPP-A expression and patient survival.

### Ethical approval and informed consent

All tissue donors provided written informed consent for tissue collection and research, which was covered by protocols approved by the Austin Health Human Research Ethics Committee, Melbourne, Australia (Approval Number H2012/04446).

## Supplementary information

Supplementary Information.

## Data Availability

All data generated or analysed during this study are included in this manuscript.
