# Supplementary material for: Aberrant pregnancy-associated plasma protein-A expression in breast cancers prognosticates clinical outcomes
Source: Sci Rep. 2020 Aug 13;10:13779. doi: 10.1038/s41598-020-70774-9 (PMC7426935; doi:10.1038/s41598-020-70774-9)
Supplement: Supplementary file 1 — Supplementary Information. [file 41598_2020_70774_MOESM1_ESM.pdf]

Supplemental Figure and Table

Aberrant Pregnancy-Associated Plasma Protein-A expression in breast cancers prognosticates clinical outcomes

Prashanth Prithviraj[1,2,3,4], Matthew Anaka[5], Erik W. Thompson[6,7], Revati Sharma[3,4], Marzena Walkiewicz[1], Candani S. A. Tutuka[1,8], Andreas Behren[1,2,8], George Kannourakis[3,4], and Aparna Jayachandran[3,9*]

[1]Olivia Newton-John Cancer Research Institute, Cancer Immunobiology Laboratory, Heidelberg, VIC, Australia.

[2]Department of Medicine, University of Melbourne, Victoria, Australia

[3]Fiona Elsey Cancer Research Institute, Ballarat, Victoria, Australia

[4]Federation University Australia, Ballarat, Victoria, Australia

[5]Department of Medicine, University of Alberta, Alberta, Canada

[6]Institute of Health and Biomedical Innovation, Queensland University of Technology (QUT), Brisbane, Australia.

[7]Translational Research Institute, Woolloongabba, Australia

[8]School of Cancer Medicine, La Trobe University, Victoria, Australia

[9]Gallipoli Medical Research Institute and The University of Queensland, Brisbane, Australia

[*]Corresponding Author:

Aparna Jayachandran, PhD

Fiona Elsey Cancer Research Institute,

Ballarat Technology Park- Central Suite 23,

106-110 Lydiard St Sth, Ballarat, VIC 3350, Australia

E-mail: aparna@fecri.org.au

Phone +61 4 2424 8058

Supplementary Table 1. Frequency of alterations in PAPP-A in the breast cancer datasets at cBioportal. As the datasets Breast Invasive Carcinoma (TCGA, Nature 2012) and Breast Invasive Carcinoma (TCGA, Cell 2015) share samples, and in turn share samples with the TCGA provision dataset which continues to have data added after the time of these publications, only results from the provision dataset are shown.

	Mutations	Copy number changes	Gene expression microarray	Gene expression RNAseq	Protein express-ion RPPA
Mutational profiles of metastatic breast cancer (France, 2016)	1 (0.5%) of 213 sequenced cases/ patients	2 (0.9%) of 213 sequenced cases/patients – 2 amplifications	N/A	N/A	N/A
Breast cancer patient xenografts (British Columbia, Nature 2014)	0 (0%) of 15 sequenced cases/ patients	N/A	N/A	N/A	N/A
Breast Invasive Carcinoma (TCGA, Provisional)	12 (1.2%) of 977 sequenced cases/ patients	10 (1%) of 977 9 amplifications, 1 deletion	26 (2.7%) of 977 – 18 up-regulation, 9 down-regulation	41 (4%) of 1093 – all up-regulation	0 (0%)
Breast Invasive Carcinoma (Sanger, Nature 2012)	2 (2%) of 100 sequenced cases/ Patients	N/A	N/A	N/A	N/A
Breast Invasive Carcinoma (Broad, Nature 2012)	1 (1%) of 103 sequenced cases/ Patients	N/A	N/A	N/A	N/A
Breast Invasive Carcinoma (British Columbia, Nature 2012)	0 (0%) of 65 sequenced cases/ patients	N/A	N/A	N/A	N/A

Breast Cancer (METABRIC, Nature 2012 &Nat Commun 2016)	0 (0%) of 2369 sequenced cases/ patients	10 (0.4%) of 2369 sequenced cases/patients – 9 amplifications, 1 deletion	68 (2.9%) of 2369 cases/patients – all upregulation	N/A	N/A

Supplemental Figure S1

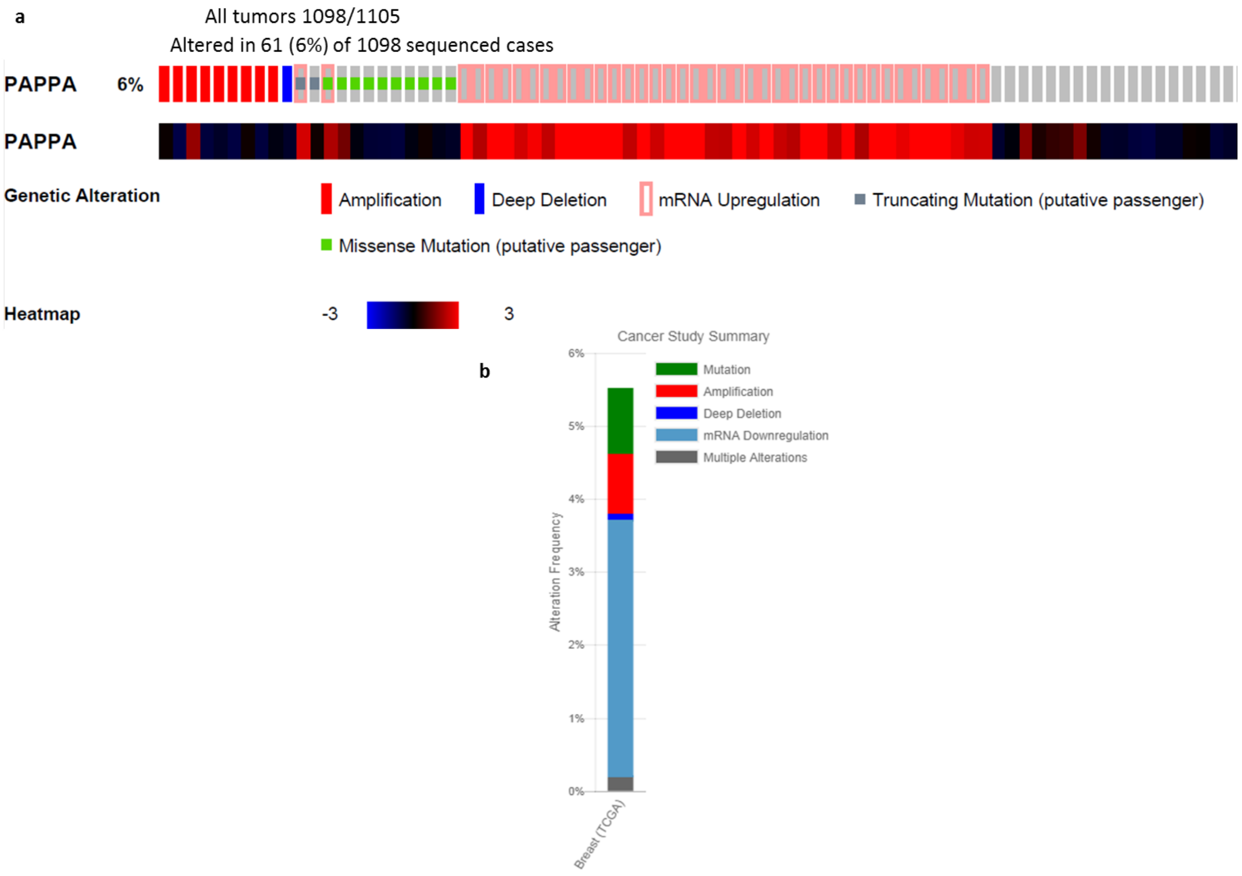

Supplemental Figure Legend

Supplementary Figure S1 (**a**) The OncoPrint from a query for alterations in expression of PAPP-A in breast cancer patients. Rows represent PAPP-A genes and heatmap, and columns represent samples. Glyphs and colour coding are used to summarize distinct genomic alterations including mutations, amplifications, homozygous deletions and changes in gene expression. By default, cases are sorted according to alterations. (**b**) Genomic alterations in breast cancer TCGA dataset is represented by colour coding.
